# Genetic Diversity among Clonal Lineages within *Escherichia coli* O157:H7 Stepwise Evolutionary Model

**DOI:** 10.3201/eid1311.070381

**Published:** 2007-11

**Authors:** Peter C.H. Feng, Steven R. Monday, David W. Lacher, Lesley Allison, Anja Siitonen, Christine Keys, Marjut Eklund, Hideki Nagano, Helge Karch, James Keen, Thomas S. Whittam

**Affiliations:** * Food and Drug Administration, College Park, Maryland, USA; † Michigan State University, East Lansing, Michigan, USA; ‡ Western General Hospital, Edinburgh, Scotland; § National Public Health Institute, Helsinki, Finland; ¶ Hokkaido Institute of Public Health, Hokkaido, Japan; # University of Munster, Munster, Germany; ** US Department of Agriculture, Clay Center, Nebraska, USA

**Keywords:** Enterohemorrhagic E. coli O157:H7, clonal complexes, genetic diversity, molecular evolution, research

## Abstract

*Escherichia coli* O157:H7 variants were examined for trait mutations and by molecular subtyping to better define clonal complexes postulated on the O157:H7 evolution model. Strains of β-glucuronidase–positive, sorbitol-negative O157:H7 isolated in United States and Japan were identical to A5 clonal strain and shared sequence type (ST)–65 by multilocus sequence typing (MLST); thus, they belong in A5. However, these strains exhibited pulsed-field gel electrophoresis (PFGE) profile differences that suggested genomic divergence between populations. Sorbitol-fermenting O157 (SFO157) strains from Finland, Scotland, and Germany were identical to A4 clonal strain and belong in A4. Some SFO157 strains, isolated years apart and from different countries, had identical PFGE profiles, suggesting a common origin. Despite similarities, some Finnish and Scottish and all of the German strains have ST-75 (“German clone”), whereas others have ST-76, a new variant (“Scottish clone”). MLST of strains in other clonal complexes also discriminated strains thought to be identical and showed that genetic differences will further distinguish clonal populations into subclones.

## Abstract 2

Molecular characterization and subtyping show genetic diversities within clonal complexes.

An evolutionary model postulates that *Escherichia coli* O157:H7 evolved from ancestral *E. coli* by stepwise acquisition or loss of virulence and phenotypic traits (*1*). At the center of the model is a hypothetical “intermediate” strain (ancestor A3), which evolved from the A2 clonal complex of O55:H7 strains that are closely related but ancestral to O157:H7 (*2*). It is hypothesized that the A3 intermediate strain is a missing link, a primitive O157:H7 that ferments sorbitol (SOR), is β-glucuronidase positive (GUD+), and has the Shiga toxin 2 gene (*stx_2_*), and from which evolved 2 distinct pathways. The loss of SOR phenotype and the acquisition of *stx_1_* gene led to the emergence of the A5 clonal complex of SOR–, GUD+ O157:H7 strains, which then lost GUD expression, resulting in the prototypic O157:H7 clonal complex (A6). Also from A3 emerged a divergent lineage caused in part by the loss of motility giving rise to the A4 clonal complex of SOR+, GUD+, nonmotile strains that are designated as SFO157. These clonal complexes on the model were predicted on the basis of phenotypes, multilocus enzyme electrophoresis (MLEE), and the presence of the +93 single nucleotide polymorphisms (SNP) in the *uid*A gene that encode for GUD (*1*). However, except for the A6 clonal complex (O157:H7), from which strains were readily available, only a few strains existed for the other clonal complexes. As a result, these clonal complexes were not well defined because of the limited characterization criteria used and the lack of strains.

Molecular typing methods have improved the ability to characterize and discriminate closely related strains. Genetic studies have also elucidated some of the mutations that occurred in the stepwise emergence of clonal complexes. For example, in the transition from A5 to A6, the loss of GUD expression was found to be due to a frame-shift mutation caused by G-G insertion at +686 in the *uid*A gene (*3*). Similarly, in the divergence of A4 from A3, the loss of motility was caused by a 12-bp deletion in the *flhC* flagella master regulon gene (*4*). These mutations provide unique markers for tracing the model’s evolutionary events and, coupled with better typing methods, have provided more discriminatory means to reexamine genetic relatedness among O157:H7 clonal complexes.

In addition, new or previously rare strains have been isolated more frequently. The A5 clonal complex of GUD+ O157:H7 was represented solely by strain G5101, isolated from a hemorrhagic colitis patient in the United States in 1994 (*5*). Similarly, the A4 clone complex of SFO157 was represented by 493–89 and a few other German strains isolated from hemolytic uremic syndrome (HUS) patients in Bavaria in 1988 (*6*). Recently, however, GUD+ O157:H7 strains have been isolated in the United States and from outbreaks and sporadic infections in Hokkaido, Japan (*7*). Likewise, SFO157 strains, which have also been isolated from cattle (*8*), are increasingly causing sporadic infections and outbreaks of HUS (*9*,*10*) and have been isolated in the Czech Republic (*11*), Finland (*12*,*13*), Scotland (*14*), and other countries (*6*).

In this study, we examined strains from various clonal complexes, including GUD+ O157:H7 strains from the United States and Japan and SFO157 strains from Finland, Scotland, and Germany, for unique mutational markers. We also used molecular typing to better define the various clonal complexes in the O157:H7 evolution model.

## Materials and Methods

### Bacterial Strains

The 3 GUD+ O157:H7 strains from the United States were originally isolated from clinical samples, and the 22 strains from Japan were isolated from outbreak patients with diarrhea or no symptoms and sporadic cases in Hokkaido (*7*). The SFO157 strains included 8 from Germany (*15*), 8 from Finland (*13*), and 5 from Scotland (*14*). Most of these were isolated from patients with symptoms of hemorrhagic colitis or HUS. However, 3 Scottish strains (designated H1085 and variations thereof) were isolated from cat feces at a farm where a hemorrhagic colitis infection had occurred. The other strains examined are from the Food and Drug Administration (FDA) and the Shiga Toxin-Producing *Escherichia coli* Center (Michigan State University, East Lansing, MI, USA), except for LSU-61, an O157:H7 strain isolated from deer (*16*).

### Characterization

To reconfirm their phenotypic traits, strains were plated on cefixime-tellurite sorbitol MacConkey agar (Bacteriological Analytical Manual, www.cfsan.fda.gov/~ebam/bam-4a.html) to test for SOR fermentation and resistance to 2.5 μg/mL potassium tellurite. The plates also had a ColiComplete disc (BioControl, Bellevue, WA, USA) to test for GUD activity. All isolates were serotyped for O157 and H7 antigens (RIM O157:H7, Remel, Lenexa, KS, USA) and tested by several PCR assays for trait virulence genes, mutational markers, and SNPs. A multiplex PCR (*17*) was used to test for *stx_1_*, *stx_2_*, *γ-eae* for intimin, *ehxA* for enterohemolysin, and the +93 SNP in the *uidA*. The GUD+ O157:H7 strains were tested for the +776 SNP and for the +686 G-G insertion in *uid*A (*3*); the SFO157 strains were tested for the presence of the H7 *fliC* gene and for the 12-bp *flhC* deletion (*4*). Shiga toxin (Stx) production was verified serologically with the Verotoxin-producing *E. coli*–Reversed Passive Latex Agglutination Test (Denka Seiken, Japan), and enterohemolysin activity was tested on Ca^++^ blood agar plates (*18*).

### Pulsed-Field Gel Electrophoresis (PFGE)

*Xba*I-digested genomic DNA was analyzed in 1% agarose gel in 0.5× Tris-boric acid-EDTA TBE buffer at 14°C by using CHEF MAPPER (BioRad, Hercules, CA, USA) (*19*). The runtime was 18 h at 6V/cm, with initial and final switch times of 2.16 and 54.17 s, respectively. The gel was stained with ethidium bromide (1 μg/mL), observed on the Gel Doc 2000 system (BioRad), and analyzed with the BioNumerics fingerprinting software (Applied Maths, St-Martens-Latem, Belgium).

### Multilocus Sequence Typing (MLST)

The MLST protocol (www.shigatox.net/cgi-bin/mlst7/index) uses PCR primers to amplify internal segments of 7 specific housekeeping genes (aspartate aminotransferase [*aspC*], caseinolytic protease [*clpX*], acyl-CoA synthetase [*fadD*], isocitrate dehydrogenase [*icdA*], lysine permease [*lysP*], malate dehydrogenase [*mdh*] and *uidA*), which are purified and sequenced. Each unique sequence is given an allele number, and the combinations of alleles from the 7 genes are used to compile the organism’s allelic profile. Each unique profile is designated as a sequence type (ST), which is then compared with those of other pathogenic *E. coli* strains in the *Ec*MLST database (*20*).

## Results

### Strain Characterizations

All the GUD+ O157:H7 strains examined had traits identical to the A5 type strain (G5101); were SOR–, GUD+, and tellurite resistant; and produced both O157 and H7 antigens (Table 1). Except for 2 US strains (TW06289 and TW06290) that did not have *stx_1_*, all carried *stx_1_*, *stx_2_*, *γ-eae*, *ehxA*, and the +93 *uidA* SNP. Serologic analysis confirmed the production of both Stx, and all strains also had enterohemolysin activity. All GUD+ O157:H7 strains had the +776 SNP and, consistent with their GUD+ phenotype, did not have the +686 *uidA* G-G insertion. Because these strains expressed H7, they were not tested for the H7 *fliC* gene or for the *flhC* deletion.

**Table 1:** Characteristics of *Escherichia coli* O157 strains*

Source	No.	SOR	GUD	Τe	O157	H7	*fliC*	*stx_1_*	*stx_2_*	*γeae*	*ehx*A	+93	+776	+686	BAP	Δ*flhC*
G5101		–	+	R	+	+	+	+	+	+	+	+	+	–	+	–
USA	1	–	+	R	+	+	ND	+	+	+	+	+	+	–	+	NA
	2	–	+	R	+	+	ND	–	+	+	+	+	+	–	+	NA
Japan	22	–	+	R	+	+	ND	+	+	+	+	+	+	–	+	NA
498/89		+	+	S	+	–	+	–	+	+	+	+	–	NA	–	+
Germany	5	+	+	S	+	–	+	–	+	+	+	+	–	NA	–	+
	2	+	+	S	+	–	+	–	–	+	+	+	–	NA	–	+
Scotland	4	+	+	S	+	–	+	–	+	+	+	+	–	NA	–	+
	1	+	+	S	+	–	+	–	+	+	–	+	–	NA	–	+
Finland	6	+	+	S	+	–	+	–	+	+	+	+	–	NA	–	+
	2	+	+	S	+	–	+	–	–	+	+	+	–	NA	–	+
LSU-61		+	+	R	+	+	+	–	–	+	+	+	–	NA	+	–

*SOR, sorbitol fermentation; GUD, β-glucuronidase activity; Te, tellurite resistant (R) or sensitive (S); O157, antigen; H7, antigen; *fliC,* H7 flagella gene; *stx_1_*, shiga toxin 1 gene; *stx_2_*, shiga toxin 2 gene; *γeae*, γ-intimin; *ehxA*, enterohemolysin gene; +93, *uid*A single nucleotide polymorphisms (SNP); +776, *uid*A SNP; +686, *uid*A G-G insertion; BAP, enterohemolysin activity on blood agar plates; Δ*flhC*, 12-bp deletion; ND, not done; NA, not applicable.

All SFO157 strains examined were SOR+, GUD+, and tellurite sensitive, and expressed the O157 but not the H7 antigen (Table 1). Despite the absence of H7, all strains carried the H7 *fliC* gene. Except for 2 German (210–89, CB1009) and 2 Finnish (IH56929, IH56776) strains that did not have *stx*, all SFO157 carried only *stx_2_*, *γ-eae*, +93 *uidA* SNP, and the 12-bp *flhC* deletion, but not the +776 *uid*A SNP. Since SFO157 strains are GUD+, they were not tested for the +686 insertion in *uidA*. Also, except for strain H1085 1a/1, all SFO157 strains carried *ehxA*, but none had enterohemolysin activity. All these traits are consistent with those of the A4 type strain (493–89).

Analysis of LSU-61 showed that it had a mix of traits from various clonal complexes. It is SOR+, GUD+, tellurite resistant, and had both O157 and H7 antigens. It had the +93 SNP but not the +776 SNP in *uidA*; had no *stx*; had *γ-eae* and *ehxA*; and showed enterohemolytic activity on blood agar plates (Table 1).

### Molecular Subtyping

**Figure 1:**
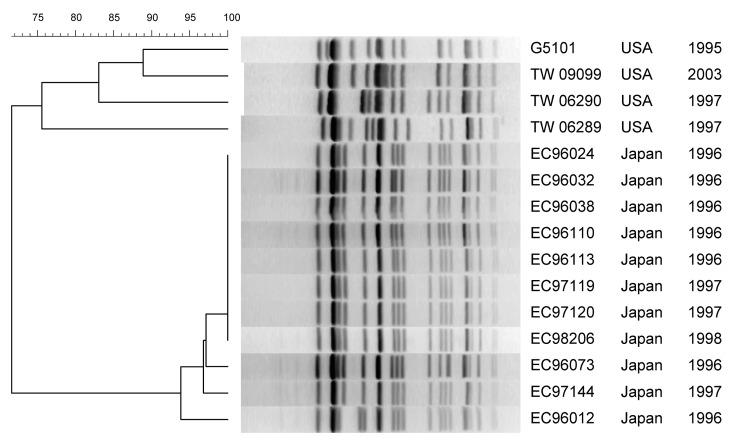
Pulsed-field gel electrophoresis of *Xba*I-digested DNA from GUD+ O157:H7 strains. Strain designation, source and year of isolation are shown at right. This unweighted pair-group method with arithmetic mean dendrogram was generated in BioNumerics software (Applied Maths, St-Martens-Latem, Belgium) by using Die coefficient with a 1.0% lane optimization and 1.0% band position tolerance. The scale above the dendrogram indicates percent similarity.

PFGE profiles of the US and 11/22 Japanese GUD+ O157:H7 strains are shown in Figure 1. The Japanese strains had nearly identical profiles, showing >95% similarity. Among the US strains, TW09099 and G5101 shared ≈90% similarity, but the other 2 strains were only 75% similar to G5101. The profiles of the US and Japanese strains shared only ≈70% similarity overall. MLST showed that all GUD+ O157:H7, including G5101, had ST-65 (Table 2).

**Table 2:** Sequence type, serotype, and isolation information of strains of *Escherichia coli*

Sequence type	Strain	Source	Year	Serotype
65*	G5101	USA	1995	O157:H7
	EC96038	Japan	1996	O157:H7
	EC96024	Japan	1996	O157:H7
	EC97144	Japan	1997	O157:H7
	TW09099	USA	2003	O157:H7
	TW06290	USA	1997	O157:H7
	TW06289	USA	1997	O157:H7
66	86–24	USA	1986	O157:H7
	93–111	USA	1993	O157:H7
	Sakai	Japan	1996	O157:H7
69	EDL-933	USA	1982	O157:H7
73	C586–65	Sri Lanka	1965	O55:H7
	TB182A	USA	1991	O55:H7
	5905	USA	1994	O55:H7
	3256–97	USA	1997	O55:H7
75	493/89	Germany	1989	O157:H–
	1782/88	Germany	1988	O157:H–
	5412/89	Germany	1989	O157:H–
	CB569	Germany	1987	O157:H–
	210/89	Germany	1989	O157:H–
	CB1009	Germany	1990	O157:H–
	514/91	Germany	1991	O157:H–
	4326/93	Germany	1993	O157:H–
	IH 53440	Finland	1997	O157:H–
	IH 56776	Finland	1998	O157:H–
	IH 57086	Finland	1999	O157:H–
	H1410	Scotland	2002	O157:H–
	IH 57225	Finland	1990s	O157:H–
76	IH 57201	Finland	1999	O157:H–
	IH 56909	Finland	1999	O157:H–
	IH 56969	Finland	1999	O157:H–
	IH 56929	Finland	1999	O157:H–
	H2687	Scotland	2003	O157:H–
	H1085C	Scotland	2003	O157:H–
	H1085 3a	Scotland	2003	O157:H–
	H1085 1a/1	Scotland	2003	O157:H–
77	ECOR-37†	USA	1970s	Ont:Hnt
237	LSU-61	USA	2001	O157:H7

*Only selected β-glucuronidase positive O157:H7 strains from Japan are shown. †Not typeable.

**Figure 2:**
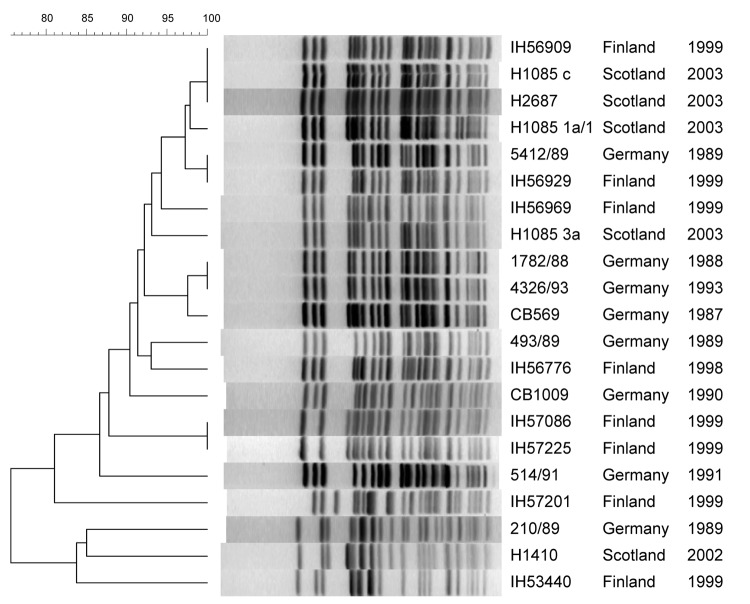
Pulsed-field gel electrophoresis of *Xba*I-digested DNA from SFO157 strains. Strain designation, source, and year of isolation are shown at right. This unweighted pair-group method with arithmetic mean dendrogram was generated in BioNumerics software (Applied Maths, St-Martens-Latem, Belgium) by using Die coefficient with a 1.0% lane optimization and 1.0% band position tolerance. The scale above the dendrogram indicates percent similarity.

The PFGE profiles of some SFO157 strains isolated within Germany (1782/88 and 4326/93) and Finland (IH57086 and IH57225) were identical (Figure 2). But profile identity was also observed among strains from Finland (IH56906) and Scotland (H1085c and H2687) and strains from Finland (IH56929) and Germany (5412/89) (Figure 2). MLST analysis showed that all the German, 4 Finnish, and 1 Scottish strain had ST-75 but that the other 4 Scottish strains and the rest of the Finnish strains had a distinct *mdh* allele and were genotyped as ST-76 (Table 2).

## Discussion

**Figure 3:**
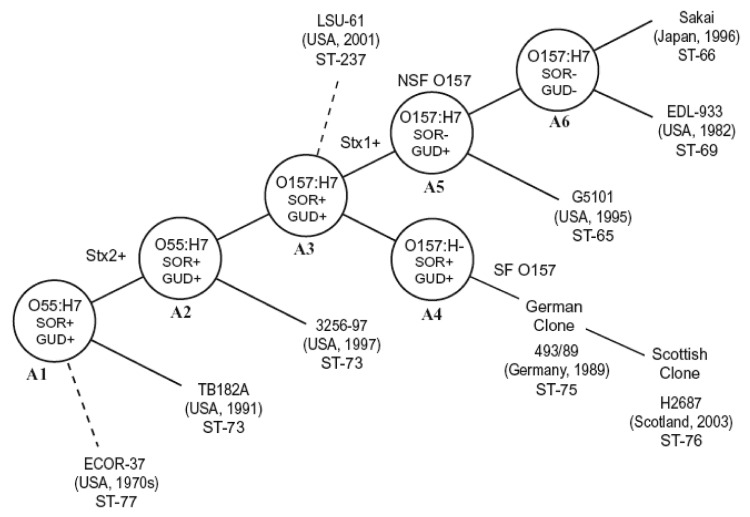
Evolution model for *Escherichia coli* O157:H7. Figure modified and updated from (*1*) to include the sequence type (ST) data showing subclones within clonal complexes. Some strains, whose position on the model remains to be determined, are shown with dashed lines.

The stepwise evolutionary model postulates that ancestral O157 clonal group (A3) split into 1 lineage, leading to the common GUD–, SOR– O157:H7 (NSF O157) clonal complex (A6) and a second branch of SFO157 that retained many primitive traits (A4) (Figure 3). The A4 and A5 clonal complexes on the evolution model are closely related to O157:H7 (A6 clonal complex) (Figure 3) and share many traits, including the +93 *uid*A SNP, which is found only in O157:H7 and its nonmotile variants (*1*). Another common trait is the γ-*eae* allele, which is also found in few other serotypes (*21*), including the O55:H7 strains in the A2 clonal complex that is ancestral to O157:H7 (*2*) (Figure 3). The A5 clonal complex of GUD+ O157:H7 strains is postulated to have emerged from the A3 intermediate strain. Analysis of A5 type strain (G5101) showed that it carried a +776 *uid*A SNP, which appears to have been acquired before the emergence of A5, because it is found only in A5 strains and A6 clonal complex of O157:H7 strains (*3*). All the GUD+ O157:H7 strains tested had identical traits as G5101, including the unique *uidA* markers (+93 and +776 SNP and absence of +686 G-G insertion) that are consistent with the mutational events postulated for the emergence of A5 and confirm that these strains also belong in A5. The fact that all these GUD+ O157:H7 strains have ST-65 supports that conclusion (Table 2). Despite similarities, however, there were differences in PFGE profiles. The Japanese strains had nearly identical PFGE profiles, which is consistent with the results by Nagano et al. (*7*), who also observed profile identity among clinical and environmental GUD+ O157:H7 isolates in Japan (*22*). In contrast, the US strains showed more diversity and shared only 70% similarity with the Japanese strains, which suggests the occurrence of recent genomic divergences among the US populations of GUD+ O157:H7 strains.

The SFO157 strains of A4 clonal complex were also postulated to have evolved from the A3 intermediate strain (Figure 3). Analysis of the A4 type strain 493–89 showed that a key mutation that led to the emergence of A4 was the loss of motility caused by the 12-bp *flh*C deletion (*4*). Other A4 traits included tellurite sensitivity and the carriage of both *ehxA* and the H7 *fliC*, neither of which is expressed. All the SFO157 strains tested had these markers, which were postulated to have been acquired in the emergence of A4, which confirmed that these SFO157 strains also belong in the A4 clonal complex. The genomic similarities of the SFO157 strains are also reflected in their PFGE profiles, as 2 German strains (1782/88 and 4326/93) isolated 5 years apart had identical profiles; a Finnish strain (IH56909) isolated in 1999 was identical to 2 Scottish strains (H1085c, H2687) that were isolated in 2003; and a Finnish strain (IH56929) was identical to a German strain (5412/89) that was isolated 10 years earlier. These results are consistent with similarities observed among SFO157 strains from Germany (*15*), Finland (*12*,*13*), and the Czech Republic (*11*). The fact that there are profile identities among strains isolated years apart and from different geographic areas suggests that these SFO157 strains isolated from various European countries may have a common origin. Despite PFGE profile similarities, MLST showed genetic differences within the SFO157 populations (Table 2). The German, 4 Finnish, and 1 Scottish strain (H1410) had ST-75, which we designated as the “German clone,” while the remaining Scottish and Finnish strains had ST-76 (“Scottish clone”) (Figure 3). This genetic difference existed among strains with identical PFGE profiles, as German strain 5412/89 and Finnish strain IH56929 had ST-75 and ST-76, respectively.

Preliminary studies show that MLST (Table 2) may also better define other clonal complexes. In the A6 complex of O157:H7 strains, the Sakai strain that caused outbreaks in Japan and 2 US strains had identical *lysP* alleles (ST-66) but were distinct from the commonly used EDL933 reference strain (ST-69), a finding that suggests that genetic differences may also further distinguish the A6 clonal complex into subclones (Figure 3).

The A1 clonal complex, which consists of O55:H7 strains that are SOR+, GUD+, and carry *γ-eae*, was postulated to have given rise to A2 by the acquisition of the *stx_2_* phage (*1*). Based on phenotypes, 2 O55:H7 strains (C586–65 and TB182A) that did not have *stx* were previously thought to be A1 strains, but MLST showed both strains to have ST-73, identical to the *Stx_2_*-producing O55:H7 strains (5905 and 3256–97) in the A2 clonal complex (Table 2). Analogous to the data obtained with the GUD+ O157:H7 strains, these O55:H7 strains had the same ST, but the PFGE profiles of C586–65 and TB182A shared only 60% similarity to 5905 and 3256–97 (data not shown). The genetic data on the transition from A1 to A2 is limited, so it is uncertain whether C586–65 and TB182A are derivatives of A1 or, perhaps, are A2 strains that have lost the *stx_2_* phage (see below).

Similarly, a marmoset isolate of *E. coli* (ECOR37) was previously shown to be closely related to A2 by MLEE. This strain had A2-like traits, including *γ-eae* (*23*), but its O and H antigens were serologically untypeable. PCR testing showed that ECOR37 had both the O55 *wzx* gene, required for export of O lipopolysaccharide, and the H7 *fliC* gene (data not shown) and, therefore, is an O55:H7 strain. However, ECOR37 shared only 60% similarity in PFGE profiles with A2 strains (data not shown). MLST also showed ECOR37 to have distinct *mdh* and *clpX* alleles (ST-77) (Table 2) and so this strain does not appear to belong in the A2 clonal complex (ST-73). We can only speculate that ECOR37 is an ancestral strain of A1 and A2, but its position in the model is uncertain (Figure 3).

LSU-61 is an O157:H7 that is SOR+; GUD+; tellurite resistant; carried *γ-eae*, *ehxA*, the +93, but not the +776 *uidA* SNP; and had no *stx*. Except for the absence of *stx_2_*, LSU-61 had many of the traits proposed for the A3 intermediate, which has not yet been isolated. If LSU-61 is the A3 intermediate strain, we would expect it to be genetically related to the other clones because A3 is thought to have evolved from A2 and to be the intermediate for both A4 and A5 (Figure 3). However, the PFGE profile of LSU-61 showed only 60% similarity to stains in the A2, A4, A5, and A6 clonal complexes (data not shown), and it had a distinct *fadD* allele (ST-237) (Table 2). Despite these dissimilarities, the fact that LSU-61 has both O157 and H7 antigens and carries traits of neighboring clonal complexes (especially +93 *uidA* SNP and *γ-eae*) is compelling evidence that it belongs in the O157:H7 complex. The absence of the +776 *uidA* SNP in LSU-61 indicates that it is ancestral to A5; however, the exact position of LSU-61 on the evolutionary model remains to be determined (Figure 3).

In our study, we encountered various strains that had identical STs and traits as the clonal type strains, except for *stx*. Both *stx_1_* and *stx_2_* are phage encoded, and there is great diversity in *stx*-phage insertion sites among strains (*24*). Sometimes, these phages may be induced, resulting in strains that have lost the ability to produce Stx (*25*) or conversely, strains may acquire the ability to produce Stx by phage infection. This acquisition or loss of *stx* phages shows that this trait may not be a stable marker. Thus, the association of *stx* genotypes with clonal complexes should be interpreted with caution.

In summary, the use of unique trait markers and molecular typing methods better defined some of the clonal complexes postulated on the O157:H7 evolution model. The GUD+ O157:H7 and the SFO157 strains obtained worldwide had the unique mutation markers postulated for the emergence of the A5 and A4 clonal types and, therefore, belonged in these respective clonal complexes. Molecular subtyping showed genetic similarities and identities among strains within clonal complexes, but MLST identified genetic differences that further segregated these strains into subclones within a clonal complex.
